# Westminster Hospital Bazaar (1905)

**Published:** 1905-05-13

**Authors:** 


### WESTMINSTER HOSPITAL BAZAAR (1905).

THE following statement has been issued in connection with the forthcoming bazaar in aid of Westminster Hospital, which is to be held in Dean's Yard, Westminster, on May 23rd and two following days.

It has ministered for nearly 200 years to the suffering inhabitants of this crowded and busy city, and the institution has been the pioneer of the voluntarily supported hospitals in London.

The ordinary annual income of the hospital falls short by £10,500 of the amount of the expenditure, and, unless this state of things can be remedied, there will be no alternative to the managers but to turn away deserving and necessitous cases from their doors; our object is, above all things, to prevent any such unfortunate catastrophe.

A "Book of the Bazaar" will be issued a few days before the date of opening, in which the details of our requirements will be explained.

May we, in the meantime, appeal to all who have such charitable institutions at heart to countenance and support this special effort which we have undertaken on behalf of this most deserving institution?—We are, &c.,

| | |
|---|---|
| GWENDOLEN NORFOLK. | HELEN M. F. BRIDGE. |
| SUSAN SOMERSET. | BESSIE GULLY. |
| LOUISA BUCCLEUCH. | MARGOT ASQUITH. |
| MILLICENT SUTHERLAND. | T. C. HENSLEY HENSON. |
| K. WESTMINSTER. | CHARLOTTE WILBERFORCE. |
| ALICE SALISBURY. | MARGARET ALLCHIN. |
| HORATIA ERSKINE. | MABEL H. CAVE. |
| DOREEN LONG. | MARY M. E. COWELL. |
| GEORGIANA LLANGATTOCK. | MARIE DICKENS. |
| WINNY ARMSTRONG. | MARY RITCHIE. |
| ISABELLE THOMAS. | MARY E. SHUTER. |
| JANIE HENDERSON. | SYLVIA M. SPOTTISWOODE. |

